# Electrohydrodynamic fabrication of core–shell PLGA nanoparticles with controlled release of cisplatin for enhanced cancer treatment

**DOI:** 10.2147/IJN.S134833

**Published:** 2017-05-23

**Authors:** Philip JT Reardon, Maryam Parhizkar, Anthony H Harker, Richard J Browning, Vessela Vassileva, Eleanor Stride, R Barbara Pedley, Mohan Edirisinghe, Jonathan C Knowles

**Affiliations:** 1 Division of Biomaterials and Tissue Engineering, UCL Eastman Dental Institute; 2 Department of Mechanical Engineering; 3 Department of Physics & Astronomy, University College London, London; 4 Institute of Biomedical Engineering, Department of Engineering Science, University of Oxford, Oxford; 5 Department of Oncology, UCL Cancer Institute, University College London, London, UK

**Keywords:** cisplatin, drug delivery, cancer chemotherapy, polymer, poly(lactic-co-glycolic acid), nanoparticles, electrohydrodynamic atomization, controlled release

## Abstract

Increasing the clinical efficacy of toxic chemotherapy drugs such as cisplatin (CDDP), via targeted drug delivery, is a key area of research in cancer treatment. In this study, CDDP-loaded poly(lactic-co-glycolic acid) (PLGA) polymeric nanoparticles (NPs) were successfully prepared using electrohydrodynamic atomization (EHDA). The configuration was varied to control the distribution of CDDP within the particles, and high encapsulation efficiency (>70%) of the drug was achieved. NPs were produced with either a core–shell (CS) or a matrix (uniform) structure. It was shown that CS NPs had the most sustained release of the 2 formulations, demonstrating a slower linear release post initial “burst” and longer duration. The role of particle architecture on the rate of drug release in vitro was confirmed by fitting the experimental data with various kinetic models. This indicated that the release process was a simple diffusion mechanism. The CS NPs were effectively internalized into the endolysosomal compartments of cancer cells and demonstrated an increased cytotoxic efficacy (concentration of a drug that gives half maximal response [EC_50_] reaching 6.2 µM) compared to free drug (EC_50_ =9 µM) and uniform CDDP-distributed NPs (EC_50_ =7.6 µM) in vitro. Thus, these experiments indicate that engineering the structure of PLGA NPs can be exploited to control both the dosage and the release characteristics for improved clinical chemotherapy treatment.

## Introduction

Platinum-based compounds such as cisplatin (CDDP) constitute one of the most widely used group of chemotherapeutic agents,^1^,^2^ employed in ~50% of cancer treatments.^3^ In a complex process, CDDP causes unrepairable platinum–DNA adducts leading to sufficient DNA damage to trigger cell apoptosis and has been shown to be highly effective in the treatment of many malignancies, including testicular, ovarian, cervical, and head and neck cancers. However, its systemic toxicity means its clinical use is dose limited, and hence, its therapeutic effect in many applications is restricted.^4^ Therefore, the development of a controlled release drug delivery system for CDDP represents a key challenge in achieving optimum clinical response for this potent chemotherapy agent.^5^

In recent years, the continued search for effective cancer treatment has led to the emergence of many nanosized vectors for the delivery of chemotherapeutics, for example, liposomes^6^ and polymeric particles,^7^,^8^ which aim to reduce premature interaction with the biological environment and improve cellular targeting.^9^,^10^ Importantly, the physicochemical properties of nanovectors influence their efficacy as drug delivery systems. Therefore, this structure–activity relationship can be harnessed to engineer nanomaterials as chemotherapeutic delivery agents for improved antitumor efficacy. It has been reported that controlling the size of particles can enable them to penetrate tissue structures.^11^ Furthermore, the structure of nanovectors strongly influences their stability and drug delivery profile. It has been shown in vitro that core–shell (CS) structures can control the release of drugs, suggesting that they have great potential as drug delivery systems.^12^,^13^ Additionally, CS structuring can enhance the thermal and chemical stability of nanoparticles (NPs) and reduce cytotoxicity.^14^

Polymeric nanosized carriers, particularly poly(lactic-co-glycolic acid) (PLGA), have been extensively reported as nanovectors due to favorable biodegradability and biocompatibility properties.^15^ Encapsulation of CDDP into polymeric NPs is a considerable challenge on account of its physicochemical properties, in particular, its poor solubility in organic solvents.^16^,^17^ Recently, electrohydrodynamic atomization (EHDA) has been utilized to successfully encapsulate both hydrophobic and hydrophilic drugs within polymeric carriers, achieving high encapsulation efficiency and exhibiting excellent control over particle size and distribution.^18^–^21^ Additionally, drug encapsulation using EHDA is a single-step procedure requiring no subsequent processing steps to remove solvents or templating agents, in effect producing delivery agents that can be directly utilized in vivo without further pretreatment.^22^ Importantly, of the various electro-encapsulation processes that have been investigated, coaxial EHDA processing holds immense potential for producing nanovector CS structures that may reduce any burst release characteristics and achieve near zero-order release kinetics.^19^,^23^

Therefore, this study aims to investigate the relationship between NP structure and antitumor efficacy to develop CDDP-loaded PLGA particles for enhanced cancer therapy. EHDA was utilized in single and coaxial configurations to form CDDP-loaded particles with varying internal structure (from uniform matrix to CS). The characteristics of the CDDP-loaded NPs were investigated systematically. First, the influence of NP structure on the delivery profile and cellular uptake was studied in vitro. Second, anticancer efficacy was examined.

## Methods

### Materials

PLGA (copolymer 50:50, Resomer RG503H, molecular weight of 33,000 Da, inherent viscosity 0.41 dL g^−1^) was obtained from Boehringer Ingelheim (Ingelheim, Germany). Dimethylacetamide (DMAc) and rhodamine B (RhB) were obtained from Sigma-Aldrich (Poole, UK). CDDP, molecular weight of 300 g mol^−1^) was purchased from Enzo Life Sciences (Exeter, UK).

### Particle fabrication

For single-needle electrospraying experiments, PLGA solutions (2 wt%) were prepared by dissolving the polymer in DMAc and mechanically stirring for 400 s. CDDP (2 mg/mL) was added to one of the solutions (for production of drug-loaded particles) followed by stirring for a further 500 s in ambient temperature (20°C) to ensure the total dissolution of both the drug and the polymer. In a separate study, 0.005 wt% RhB was added to both solutions (with and without CDDP) to evaluate uptake of the NPs into tumor cells.

In order to prepare CS particles with the coaxial configuration, 2 mg/mL of CDDP was dissolved in DMAc as the inner (core) solution. PLGA (2 wt%) was dissolved in the same solvent (DMAc) to prepare the outer (shell) solution. For the purpose of the cell uptake studies, an additional solution containing 0.005 wt% RhB and 2 wt% PLGA was prepared to label the outer shell of the particles prepared with the coaxial configuration.

The solutions were electrosprayed using both single-needle EHDA and coaxial setups (Figure S1) to produce particles. In the single-needle configuration, the solutions were made to flow through a stainless steel needle (18 G, inner diameter [ID]: 0.84 mm and outer diameter [OD]: 1.27 mm) via a syringe pump (PHD 4400; Harvard Apparatus Limited, Edenbridge, UK) at a constant flow rate of 2.5, 3, 4, or 5 µL/min. For the coaxial configuration, the inner drug and outer polymer solutions were fed through coaxial stainless steel needles of 19 G (ID: 0.69 mm and OD: 1.07 mm) and 16 G, (ID: 1.2 mm and OD: 1.6 mm), respectively. The flow rates adjusted for these solutions in order to achieve a stable cone jet were 2 and 4 µL/min for inner and outer solutions, respectively. A high precision voltage supply (Glassman Europe Ltd, Bramley, UK) was used to apply an electric potential difference between the needle and a ground electrode to the solution and was varied from 12 to 20 kV as required to form a stable cone jet. The particles were collected at a working distance of 200 mm below the device exit directly on to glass slides or aluminum foil, respectively, for characterization and measurement of drug release. The jet and particle formation processes were monitored using a Leica DMS300 camera (Leica Microsystems, Wetzlar, Germany). Experiments were conducted at the ambient temperature of 19°C–21°C and relative humidity of 40%–50%. Each experiment was conducted at least 3 times to ensure the reproducibility of the EHDA process and consistency of the particles produced.

### Particle characterization

#### Optical microscopy and scanning electron microscopy

Samples of particles were collected on glass slides. These were analyzed initially under an optical microscope (Eclipse ME 600; Nikon, Tokyo, Japan) fitted with a camera (Micropublisher 3.3 RTV, 3.3 megapixel CCD Color-Bayer Mosaic, Real Time Viewing camera; Media Cybernetics, Marlow, UK). Further analysis of particle size and morphology was carried out using a field emission scanning electron microscope (SEM; XL30 FEG; Philips, Amsterdam, the Netherlands). Both optical (bright-field) and scanning electron micrographs were analyzed using ImageJ to determine the average diameter and standard deviation of the population of particles (300 particles were measured from each sample). An INCA X-sight EDAX system (Oxford Instruments, Abingdon, UK) was used with the XL30 microscope for energy dispersive X-ray (EDX) spectroscopy analysis to identify the presence of CDDP in the NPs.

#### Fourier transform infrared spectroscopy

The infrared spectra of CDDP, PLGA NPs, and drug-loaded NPs were recorded using a Fourier Transform Infrared (FTIR-ATR-Perkin Elmer 2000, PerkinElmer, Waltham, MA, USA) spectrophotometer. Spectra of all materials were recorded using a frequency range of 400–4,000 cm^−1^ and averaged over 4 runs. Powdered samples were placed on the attenuated total reflectance (ATR) crystal and then compressed using an axial screw.

#### Transmission electron microscopy

The structural characteristics of the NP formulations and CDDP distribution within them were examined using a transmission electron microscope (TEM; CM12; Philips) and EDX analysis (JEM-2100 – Jeol TEM fitted with X-max EDAX system – Oxford Instruments). For this part of the work, particles were sprayed directly on to carbon-coated copper grids and analyzed without additional contrast.

### In vitro drug release

Following a previously published protocol,^24^ 20 mg of CDDP-loaded NPs were dispersed in 1.5 mL of phosphate-buffered saline (PBS) (pH 7.4) and incubated at 37°C. At predetermined time intervals, the dispersion was centrifuged (14,000 rpm), and then 0.5 mL aliquots of solution were removed for the purpose of measurement and replaced with fresh buffer solution. Aliquots of the supernatant were centrifuged and analyzed using a VP Series High Performance Liquid Chromatography (HPLC) system (Shimadzu, Kyoto, Japan). Liquid chromatography was optimized on a 250×4.6 mm diameter Hypersil GOLD SAX column (ThermoFisher Scientific, Waltham, MA, USA) under isocratic conditions using a UV/VIS photodiode array detector (Shimadzu). A mobile phase of NaCl solution (0.9% w/v) and methanol (95:5) was used at a flow rate of 0.7 mL/min. A calibration plot to calculate the CDDP concentrations of unknown measurements was produced by measuring the absorbance of different concentrations of CDDP solution (0.9% w/v saline) from 1 to 50 µg/mL.

In order to determine the encapsulation efficiency of CDDP, 10 mg of CDDP-loaded nano/microparticles were mixed with DMAc followed by addition of PBS. The solution was then passed through a 0.22 µm filter and analyzed by reverse-phase HPLC method (refer “In vitro drug release” section). Encapsulation efficiency (percentage of the amount of drug added initially that was entrapped in the NPs) was calculated using the formula:

Encapsulation efficiency(EE%)=Wt/Wi×100% (1)

where Wt is the actual drug loading and Wi is the weight of drug used in particle synthesis.

The NonlinearModelFit function within Mathematica (Wolfram Research, Champaign, IL, USA) was used to fit the release rates of uniform and CS CDDP-loaded PLGA particles. Quality of fit was assessed using the adjusted *R*^2^ parameter (the adjustment allows for different numbers of fitting parameters).

### In vitro cell cytotoxicity assays

Cell viability was determined in a human head and neck squamous carcinoma cell line (UM-SCC-47) obtained from Dr Thomas Carey, University of Michigan. All the UM-SCC cell lines were established from head and neck cancer patients who gave written informed consent in studies reviewed and approved by the University of Michigan Medical School Institutional Review Board and by the UCL Cancer Institute for their use in this study. UM-SCC-47 cells were grown in Roswell Park Memorial Institute (RPMI)-1640 medium supplemented with 10% fetal bovine serum (FBS).

For the 3-(4,5-dimethylthiazol-2-yl)-2,5-diphenyltetrazolium bromide (MTT) assay (Sigma-Aldrich, St. Louis, MO, USA), cultured UM-SCC-47 cells were seeded into 96-well flat-bottomed plates at a density of 5×10^3^ cells in 100 µL of medium and incubated for 24 h in a 5% CO_2_ atmosphere at 37°C. They were then incubated in growth medium containing different concentrations of CDDP or equivalent CDDP-loaded PLGA NPs for 24, 48, or 72 h. Media containing different CDDP dosages were made up from successive dilutions in warmed media, from a stock solution of CDDP in sterile PBS (1 mM). After treatment, MTT (5 mg/mL in PBS) was diluted 1:100 with medium into each well. After 2 h of incubation, culture supernatants were aspirated, and purple insoluble MTT product was dissolved in 100 µL of dimethyl sulfoxide (DMSO)/ethanol (EtOH) (50:50) for 10 min. The absorbance in each well was recorded at 570 nm using a microplate reader; blanks were subtracted from all data, and the results were analyzed using Origin software (OriginLab, Northampton, MA, USA). Viability was presented as the percentage of the absorbance of CDDP-treated cells to the absorbance of nontreated cells as a function of CDDP concentration.

In the live/dead assay, UM-SCC-47 cells were seeded on glass coverslips in 24-well plates, 50,000 cells per well. When cells reached 70% confluency, they were treated with growth medium containing different concentrations of CDDP or equivalent CDDP-loaded PLGA NPs for 24, 48, or 72 h. Live and dead cells were then separately stained. Briefly, each well was washed with PBS twice before 1 mL of 2 µM calcein AM (ThermoFisher Scientific) and 4 µM ethidium homodimer-1 (ThermoFisher Scientific) working solution was added to each well and incubated for 40 min at ambient temperature. Finally, each coverslip was mounted on a glass slide and viewed by confocal microscopy (radiance 2100 laser scanning confocal microscope; Bio-Rad, Hercules, CA, USA) attached to a BX51 microscope (Olympus, Tokyo, Japan).

### Fluorescence-activated cell sorting analysis for apoptosis

Cells were grown in 24-well plates incubated in the presence of CDDP-loaded PLGA NP or free CDDP at 37°C for 24, 48, and 72 h. After the treatment period, the cells were removed and washed thrice with PBS. The cells were then treated with annexin-V (Invitrogen, Carlsbad, CA, USA), a marker of apoptosis and incubated in the dark, at ambient temperature, for 15 min. The cells were then washed with PBS and incubated with propidium iodide (PI) solution. The cell suspensions were then transferred to fluorescence-activated cell sorting tubes and analyzed using a Coulter Epics XL instrument.

### UM-SCC-47 cellular uptake studies

UM-SCC-47 cells were seeded in 24-well plates at a density of 50,000 cells per well, and cultured in RPMI-1640. At near confluency, the medium was removed, and the cells were washed twice with PBS. Cells were then exposed to PLGA NPs labeled with RhB at a concentration of 10 µM in growth medium for 24 h. After 24 h of incubation, the cells were washed twice with PBS, detached using trypsin– EDTA, and the amount of label associated with the cells was assayed by fluorescence measurements (λ excitation: 553 nm, λ emission: 627 nm).

The uptake of NPs by the UM-SCC-47 cells was also examined by confocal microscopy. UM-SCC-47 cells were seeded on glass coverslips in 24-well plates, 50,000 cells per well. When cells reached 70% confluency, they were treated with CDDP-loaded NPs that were labeled with RhB for 24 h. The cells were then fixed with 4% paraformaldehyde for 20 min at ambient temperature and washed twice with PBS and stained with phalloidin to visualize the actin cytoskeleton. Alternatively, for colocalization studies, the cells were washed with PBS and incubated with LysoTracker-Green (New England Biolabs). Images were obtained using a BioRad radiance 2100 laser scanning confocal microscope equipped with green and red filters.

## Results and discussion

### Characteristics of the NPs

**Figure 1:**
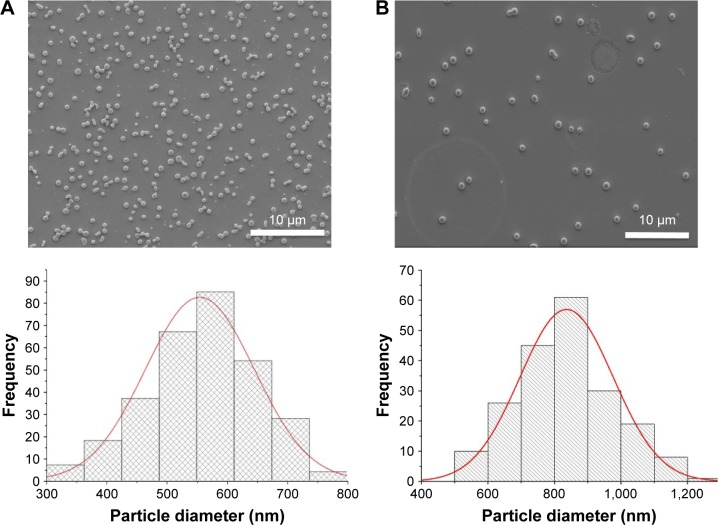
Scanning electron microscope and corresponding size distribution graphs of (**A**) U-CDDP and (**B**) CS-CDDP nanoparticles produced by single- and coaxial needle EHDA configurations, respectively. **Abbreviations:** U, uniform; CS, core–shell; CDDP, cisplatin; EHDA, electrohydrodynamic atomization.

**Figure 2:**
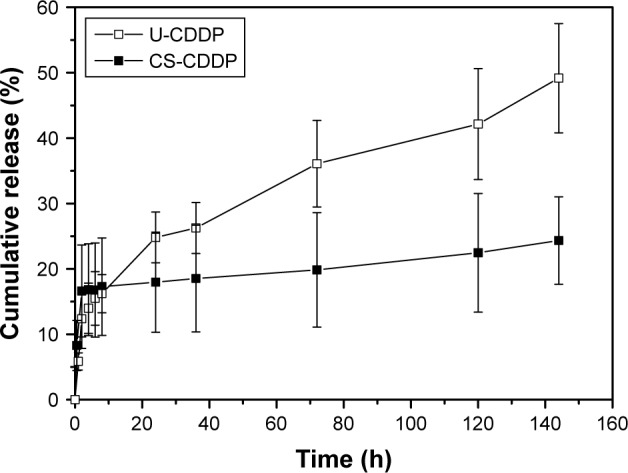
Structure-dependent CDDP release. **Notes:** PLGA nanoparticles with U-CDDP and CS-CDDP loading were incubated in PBS at 37°C and pH 7.4. The data show the mean ± SD (n=3). **Abbreviations:** PLGA, poly(lactic-co-glycolic acid); U, uniform; CS, core–shell; CDDP, cisplatin; PBS, phosphate-buffered saline; SD, standard deviation.

Using a single-needle EHDA method, with a loading efficiency >70%, the mean diameter of NPs varied with the amount of loaded CDDP, as previously reported,^21^ and the higher CDDP loading of 10 wt% (U-CDDP) induced the smallest average diameter of 550±80 nm (Figure 1). An initial burst in release was observed within the first 4 h (14% of drug was released for 10 wt%), followed by a sustained release profile (Figure 2), indicating the potential of CDDP-loaded PLGA NPs as a controlled drug delivery system.

**Figure 3:**
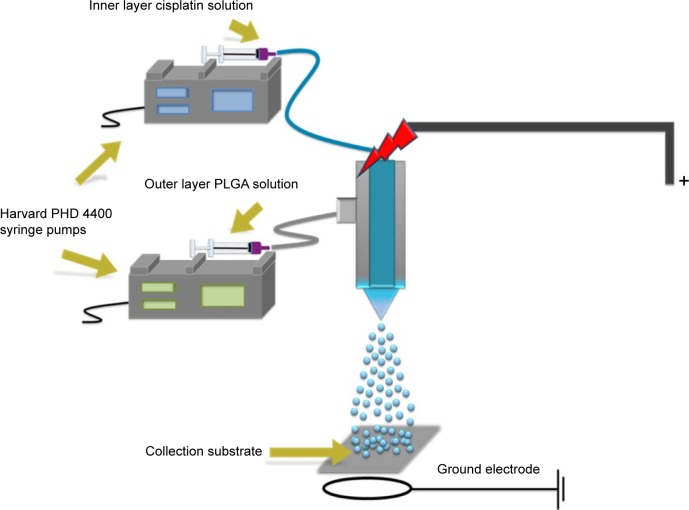
Schematic of the experimental setup used to prepare CS-CDDP drug-loaded particles. **Abbreviations:** CS, core–shell; CDDP, cisplatin; PLGA, poly(lactic-co-glycolic acid).

However, since the drug release profile plays a potentially crucial role in the efficacy of a chemotherapy treatment, we sought to further control this process by developing a coaxial needle EHDA methodology to produce a CS-type CDDP PLGA NP (Figure 3). As shown in Figure 1, novel CS-CDDP (CS-CDDP) NPs were produced with a smooth outer surface and a mean diameter of 850±200 nm. Furthermore, high encapsulation efficiencies (>80%) were achieved in agreement with data for coaxial electrospraying of other chemotherapeutics.^25^

**Figure 4:**
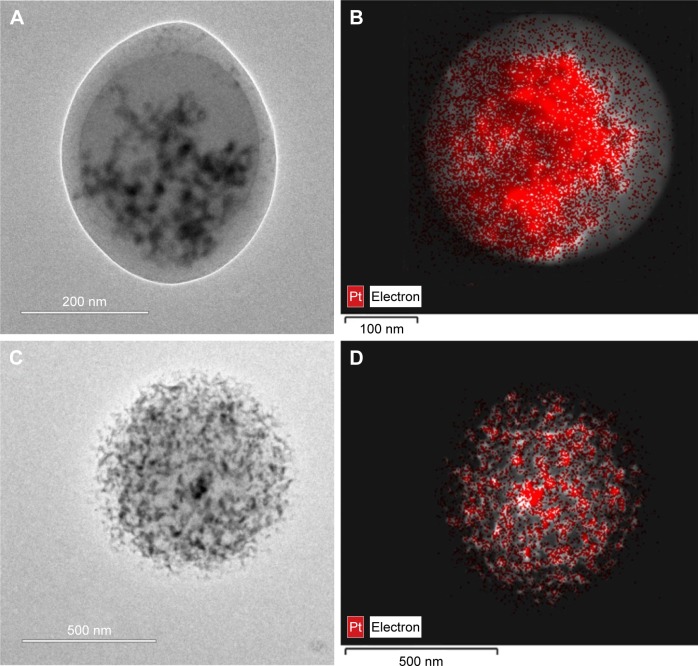
TEM bright-field micrographs of (**A**) CS-CDDP and (**C**) U-CDDP NPs and dark-field STEM micrographs of (**B**) CS-CDDP and (**D**) U-CDDP NPs with overlaid EDS Pt mapping (red). **Abbreviations:** TEM, transmission electron microscope; CS, core–shell; U, uniform; CDDP, cisplatin; NPs, nanoparticles; STEM, scanning transmission electron microscope; EDS, energy-dispersive X-ray spectroscopy; Pt, platinum.

The drug distribution within NPs potentially plays an important role in controlling delivery; therefore, we sought to examine the CDDP within the loaded NPs. FTIR spectra of PLGA NPs loaded with CDDP are shown in Figure S1. All the NPs showed characteristic PLGA peaks attributable to C=O stretching (1,754 cm^−1^) and C–O stretching (1,050–1,250 cm^−1^) and a weak peak for amine stretching (3,294 cm^−1^), indicating the presence of intact drug (CDDP), in line with EDS spectra (Figure S2). Additionally, the thermogram of the CDDP-loaded materials (Figure S3) did not show an exothermic peak at 281°C, observed for the drug alone, suggesting that the drug is molecularly dispersed in an amorphous form.^26^ Energy-dispersive X-ray spectroscopy (EDS) Pt mapping was used to evaluate the distribution of CDDP within both types of NPs. It can be seen that the drug is well distributed throughout the single-needle-derived U-CDDP NPs (Figure 4). In contrast, importantly, distribution of the drug in the coaxial needle-derived CS-CDDP particles is more localized to the core of the particles, concurrent with variation in intensity observed in the TEM micrographs, suggesting a CS structure (Figure 4A).

### In vitro drug release characteristics

It can be seen in Figure 2 that a biphasic release profile was observed under physiological conditions for both the single-needle (U-CDDP) and the coaxial (CS-CDDP) formulations. Among the previously reported single-needle NP formulations with different quantities of CDDP, we selected 10 wt% of CDDP-loaded PLGA NPs for further in vitro and in vivo experiments, because these NPs showed a sufficiently high loading amount and the best sustained release (smallest burst release – 14%).^21^

However, the CS-CDDP formulation demonstrated a shorter burst release period (~1 h), followed by a controlled zero-order (linear) release period. The higher linearity of the second release stage of the CS-CDDP NPs in comparison to the U-CDDP NPs can be explained by the different architecture of the CS-CDDP particles. The CS-CDDP NPs have a region of lower CDDP density around the perimeter of the particles. As expected, the CDDP release rate from the CS-CDDP NPs is slower than that of the U-CDDP NPs, due to the presence of the PLGA shell through which the drug is released. This structural feature is potentially favorable for controlled release of drug molecules at the tumor site, while serving as an inhibitor against leaking of the CDDP-loaded NPs. This is consistent with previously reported CS structured particles produced by coaxial electrospraying that often exhibit a sustained drug release that is tunable by adjusting the shell material or thickness.^25^

The underlying processes in drug release from PLGA are quite complex,^27^ involving diffusion of water and drug and hydrolysis and structural modification of PLGA. It is known, however,^28^ that the timescale for significant degradation of PLGA in water, although composition-dependent, is at least a month. It is therefore reasonable to assume that the release rates in Figure 2 should be described, after the initial burst, by diffusion through material with a constant diffusion coefficient. Analytical results for which are well known for a solid sphere and a spherical shell.^28^

The U-CDDP NP release in Figure 2 can be modeled by assuming a uniform distribution of CDDP in a PLGA matrix with a 550 nm diameter and a diffusion coefficient *D* = 4 1.×10^−21^m^2^s^−1^. This was determined by choosing a value of *D* that gave the best fit to the release kinetics (a goodness-of-fit parameter *R*^2^ of 0.9795 was achieved), using the expression for release fraction *φ* from particles of radius *a* as a function of time *t* derived by Eltayeb et al:^29^

$$\varphi \left(t\right)=\tanh\left(\frac{6{\left(Dt\right)}^{1/2}}{\pi^{1/2}a}\right)$$ (2)

Since the polymer structure should be similar, we assume that the same value of *D* applies to the CS-CDDP NPs. The release rate from these particles is very nearly linear, and according to the theory of spherical shell diffusion, the deviation from linear behavior involves a series of terms involving^30^,^31^

$$\exp\left[-\frac{Dn^{2}\pi t}{{\left(b-a\right)}^{2}}\right]$$ (3)

where *t* is the time, (*b* − *a*), the difference between the particle radius and the core radius, is the shell thickness, and *n* is an integer ≥1.

It is clear from the release kinetics in Figure 2 that any deviation from linear behavior occurs on a timescale <2 h, requiring a shell thickness of 50 nm or less, consistent with observations made from TEM micrographs of this material (Figure 4). For small releases from the core, the fraction release in the linear regime is

$$\varphi \left(t\right)=\frac{3bDt}{a^{2}\left(b-a\right)P}$$ (4)

where *P* is the partition coefficient for CDDP, giving the ratio of the concentration in the core to the concentration at the inner surface of the shell.

Using a shell thickness of 50 nm and particle diameter of 850 nm, fitting equation 4 to the near-linear part of the release rate at times in excess of 1 h gives a fractional release rate of 0.052% h^−1^ with an *R*^2^ value of 0.9. This leads to a partition coefficient of 5.2, comparable with the measured partition coefficient of CDDP between CH_2_Cl_2_ (a proxy for a lipid shell) and water, which was 16.3.^32^ PLGA is more hydrophilic than CH_2_Cl_2_, so it is reasonable that our partition coefficient is smaller. Therefore, we conclude that the release profiles shown in Figure 2 are consistent with simple diffusive release.

### Characterizing the efficacy of NPs in vitro

Both the prepared drug delivery NPs are expected to act as an intracellular depot increasing CDDP efficacy. Rapid dissociation of the drug from NPs may result in premature release in the blood stream, significantly reducing the efficient delivery of the drug molecules to the desired tissue/organ and increasing systemic toxicity, hence limiting dosage. Therefore, it is crucial to both control the delivery profile of drugs from NPs and evaluate its effect in promoting improved chemotherapy.

#### In vitro activity of CDDP-loaded NPs on squamous carcinoma cell line

**Figure 5:**
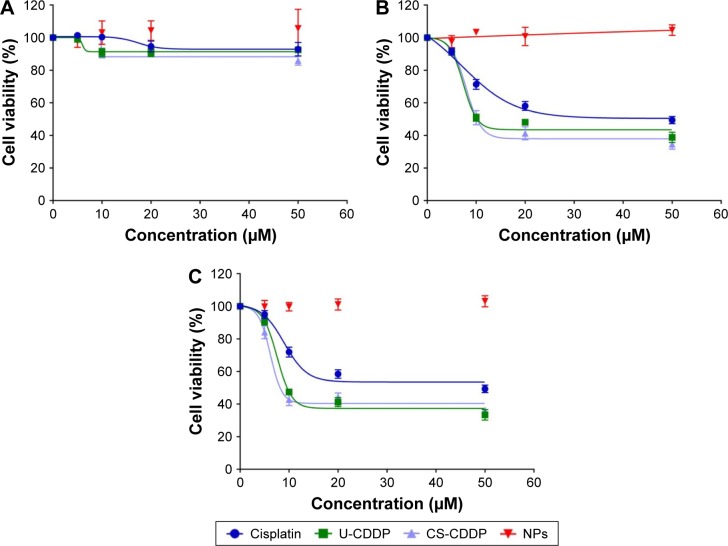
In vitro cytotoxicity of different dosages (5–50 µM) of free CDDP, CDDP-PLGA NPs, and equivalent non-drug-loaded NPs against UM-SCC-47 cells after incubation for (**A**) 24 h, (**B**) 48 h, and (**C**) 72 h. **Note:** The results represent the mean ± SD (n=6). **Abbreviations:** PLGA, poly(lactic-co-glycolic acid); NPs, nanoparticles; SD, standard deviation; U, uniform; CS, core–shell; CDDP, cisplatin.

The cytotoxic of free CDDP and CDDP-loaded PLGA NPs was evaluated in vitro by viability assay (MTT colorimetric assay), using a human head and neck squamous carcinoma UM-SCC-47 cell line, and results are expressed as % relative cell viability (Figure 5). The CDDP dosages were selected based upon the measured 50% maximal response (concentration of a drug that gives half maximal response [EC_50_]) values for free CDDP.

As shown in Figure 5, blank PLGA NPs with 0 wt% CDDP loading exhibited negligible toxicity against UM-SCC-47 cells at all time points, consistent with the high biocompatibility of this material.^33^

**Figure 6:**
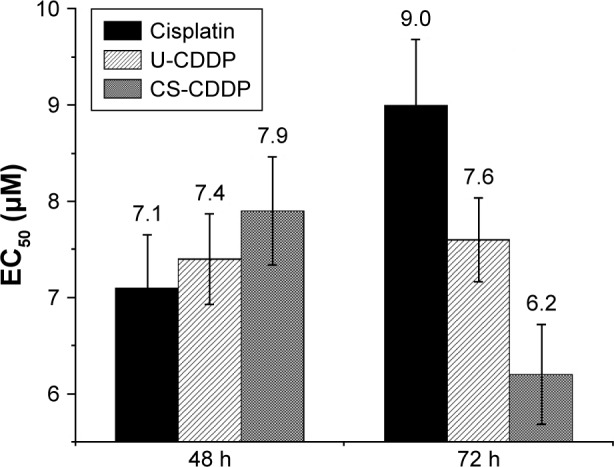
EC_50_ values for free CDDP and CDDP-PLGA NPs after incubation for 48 h and 72 h. **Note:** The results represent the mean ± SD (n=6). **Abbreviations:** PLGA, poly(lactic-co-glycolic acid); NPs, nanoparticles; SD, standard deviation; U, uniform; CS, core–shell; CDDP, cisplatin; EC_50_, concentration of a drug that gives half-maximal response.

When the UM-SCC-47 cells were exposed to the NPs loaded with CDDP for 24 h, the particles exhibited an in vitro anticancer activity similar to free drug at the same dosage. After 48 h, there was a large decrease in cell viability for both the NP groups (EC_50_ =8 and 7.4 µM for U-CDDP and CS-CDDP, respectively), for example, this dropped from ~90% at 24 h to 50% at 48 h for the 10 µM dosage. Similarly, the EC_50_ for the free drug at 48 h dropped to 7.1 µM. Importantly, the UM-SCC-47 cells exposed to 10 µM CDDP NPs for 72 h displayed a further drop in viability to ~40% (EC_50_ =7.6 and 6.2 µM for U-CDDP and CS-CDDP, respectively; Figure 6) compared to 70% and 45% for doses of 10 and 20 µM free CDDP, respectively (EC_50_ =9.0 µM), indicating potential enhancement in anticancer activity from the NP formulations. Furthermore, it is interesting to note that at the 72-h time point, only ~35% and 20% of the CDDP is released from the U-CDDP and CS-CDDP NPs, respectively (Figure 2).

**Figure 7:**
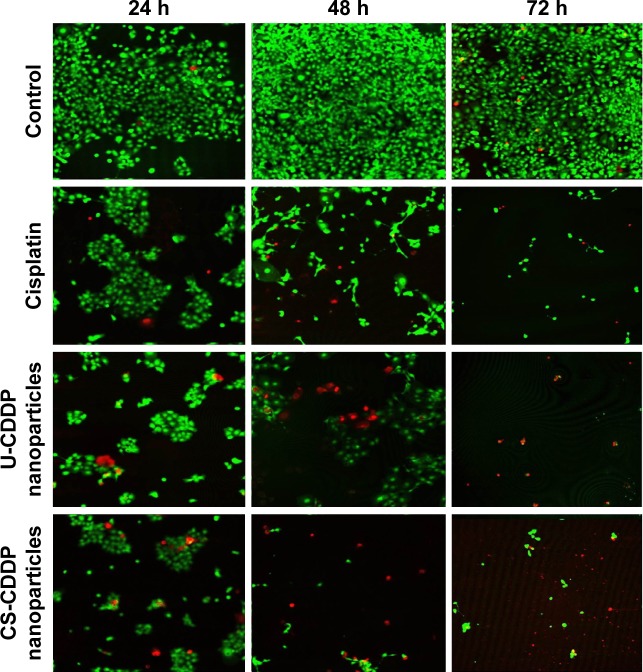
Live/dead cell staining for UM-SCC-47 cells with treatment of media (control), U-CDDP NPs (10 µM), and CS-CDDP NPs (10 µM). **Note:** Live cells were stained green with calcein-AM and dead cells were stained red with ethidium homodimer-1. **Abbreviations:** U, uniform; NPs, nanoparticles; CS, core–shell; CDDP, cisplatin.

The temporal viability of UM-SCC-47 cells was also observed by staining live and dead cells with calcein-AM and ethidium homodimer-1, respectively. Consistent with MTT results, there is a dramatic reduction in the number of live (viable) cells after 72 h treatment with both the free drug and CDDP-loaded NP formulations, in contrast to the control group that exhibited cell growth (Figure 7). This cell killing was highest after 48 h and is particularly marked for the NP groups, concurrent with the increased anticancer efficacy observed in the MTT experiments. Assuming the release of CDDP plays the crucial role in cytotoxicity of the CDDP-PLGA particles, this indicates that both types of NPs enhance the anticancer efficacy of CDDP, consistent with a sustained release profile that potentially achieves a metronomic dosing effect (equally spaced administration of lower doses of chemotherapeutic drugs without extended rest periods). This treatment has shown potential for increased antitumor efficacy with very low toxicity.^34^

**Figure 8:**
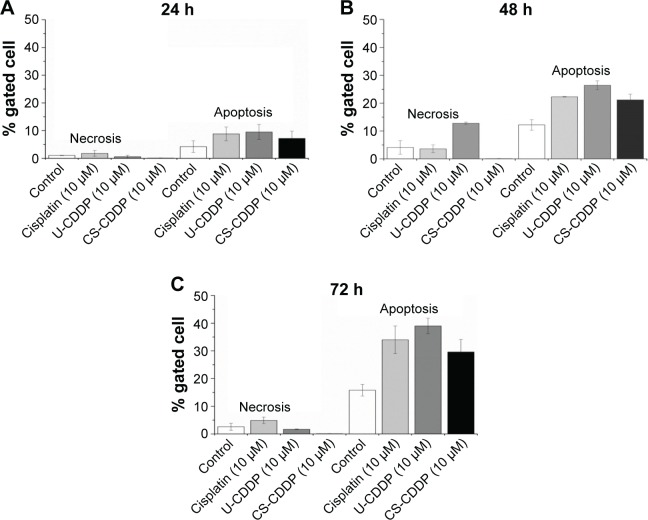
FACS data showing percentage of cells at each stage of death following different treatments measured at 24 h (**A**), 48 h (**B**), and 72 h (**C**) time points. **Abbreviations:** FACS, fluorescence-activated cell sorting; U, uniform; CS, core–shell; CDDP, cisplatin.

To further evaluate anticancer efficacy and the mechanism of cell death, we labeled the treated and non-treated cells with FITC-labeled Annexin V, which binds to phosphatidylserine exposed on the outside of the cell membrane in apoptotic cells. The cells were counterstained with PI that labels the nucleus of late apoptotic and necrotic cells. As seen in Figure 8, treatment with both CDDP-loaded NPs and free CDDP induces comparable apoptosis of the tumor cells. After 48 h, there was an increase in necrosis and apoptosis for both the free drug and NP treatments. The increase was slightly greater for the U-CDDP NP treatment, from ~10% apoptosis (24 h) to 27% (48 h), compared to 22% and 20% (48 h) for free drug and CS-CDDP, respectively. Interestingly, the necrosis level was also highest for the U-CDDP NPs, which could be due to the greater rate of CDDP release by the NPs with uniform CDDP distribution (U-CDDP) compared with the CS-CDDP formulation (Figure 8). There was a small further increase in apoptosis to ~34%, 39%, and 30% for free, U-CDDP, and CS-CDDP encapsulated CDDP, respectively, at the 72-h time point, correlating with results from cell viability studies (Figures 5–7). Therefore, treatment with the synthesized CDDP-loaded NPs can effectively induce apoptotic cell death, implying that the cytotoxicity of the drug-loaded particles is due to a sustained delivery of active CDDP. Furthermore, the dosage and hence the delivery period of CDDP are important in controlling cell death.

### Characterizing the uptake of NPs in vitro

To evaluate uptake into tumor cells, we labeled the NPs by complexing PLGA with RhB. After 24 h incubation with 10 µM equivalent of CDDP-loaded NPs, UM-SCC-47 cells demonstrated similar fluorescence intensity using a fluorimeter for both the CS-CDDP and the U-CDDP NPs, indicating no significant differences in internalization between the 2 particle architectures. This is in agreement with particle characterizations (Figures 1, 4, and S1), which revealed their relatively similar chemical composition and size.

**Figure 9:**
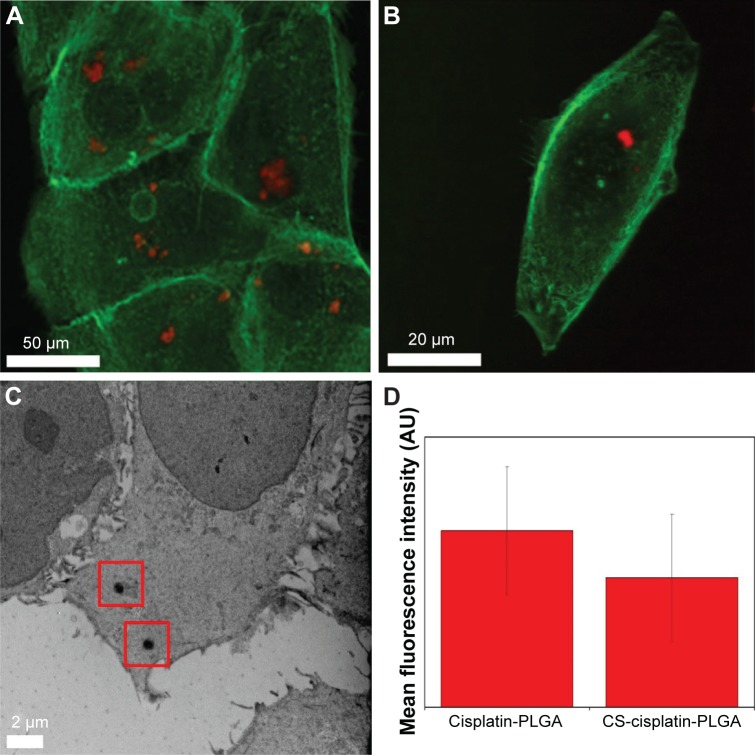
(**A** and **B**) Confocal microscope images and (**C**) TEM micrograph of UM-SCC-47 cells after interaction with (**A** and **C**) U-CDDP and (**B**) CS-CDDP NPs. (**D**) Cellular uptake expressed as mean fluorescence intensity. **Note:** Particles were functionalized with rhodamine B. Red squares indicate the location of particles within the cell (**C**). **Abbreviations:** AU, arbitrary unit; TEM, transmission electron microscope; U, uniform; CS, core–shell; CDDP, cisplatin; NPs, nanoparticles; PLGA, poly(lactic-co-glycolic acid).

Visual evidence of NP uptake by the UM-SCC-47 cells was also obtained by confocal microscopy, after incubation for 24 h. As shown in Figure 9, significant internalization was observed as intense fluorescence (red) of NPs inside the cytoplasm of the cells. In control experiments with non-RhB-labeled particles, no fluorescence was observed.

**Figure 10:**
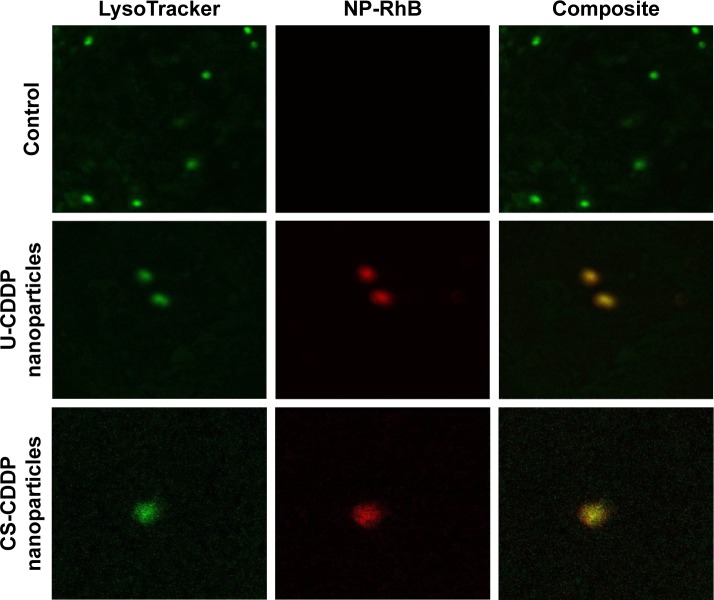
Confocal images showing examples of typical uptake of RhB-labeled PLGA NPs (red). **Notes:** The cells were counterstained with LysoTracker-Green (green) to highlight endolysosomal compartments; colocalization of the signals shows internalization of the NPs into the endolysosomal compartments. **Abbreviations:** RhB, rhodamine B; PLGA, poly(lactic-co-glycolic acid); NP, nanoparticle; U, uniform; CS, core–shell; CDDP, cisplatin.

The particle internalization was further evaluated by co-labeling the cells with a LysoTracker-Green dye to identify the endolysosomal compartments. As shown in Figure 10, NPs appear to be internalized into endolysosomal compartments. It has been suggested that internalization of CDDP-loaded carriers is a key factor in increasing the drug’s efficacy.^35^,^36^ Therefore, the enhanced cytotoxicity of the PLGA nanocarriers in comparison to free drug may also be explained by the effective endocytosis exhibited by both types of NPs. The CS-CDDP NPs demonstrated relatively similar cytotoxicity to the U-CDDP NPs, although they released ~10% less drug over the 72 h period, possibly, because they released less of the drug into the cell medium and more intra-cellularly. Clearly, a combination of controlled release and efficient cellular uptake is required to increase the efficacy of this chemotherapeutic. Importantly, reducing the proportion of CDDP delivered prematurely is also crucial for reducing systemic toxicity associated with this drug in vivo.

## Conclusion

We have demonstrated that the architecture of drug-loaded NPs can be engineered using rational design of an electrohydrodynamic system, which improves anticancer efficacy of CDDP. Novel CS NPs demonstrated a shorter burst release and slower controlled release of CDDP, yet achieved similar cancer cell cytotoxicity to the uniform CDDP-distributed particles, which was also greater than free drug. Having shown the potential for increasing the maximal tolerated dose of this potent and widely employed, yet highly nephrotoxic, chemotherapeutic agent in vitro, these findings will be further confirmed with additional cell lines and by future in vivo experiments. The ubiquitous clinical use of CDDP, and widely demonstrated biocompatibility of PLGA, should facilitate translation of this technology and indicates that architecturally engineered delivery systems of this type should serve as an effective platform for successful cancer therapy.

## Supplementary materials

Figure S1 FTIR spectra of (**A**) CDDP, (**B**) U-CDDP, (**C**) CS-CDDP, and (**D**) PLGA.
**Abbreviations:** AU, arbitrary unit; FTIR, Fourier transform infrared; U, uniform; CS, core–shell; CDDP, cisplatin; PLGA, poly(lactic-co-glycolic acid).

Figure S2 Typical EDS spectra of CDDP region of CS-CDDP NPs.
**Abbreviations:** EDS, energy-dispersive X-ray spectroscopy; CS, core–shell; CDDP, cisplatin; NPs, nanoparticles; At%, atomic percent (the percentage of one kind of atom relative to the total number of atoms).

Figure S3 DSC thermogram of (**A**) CDDP, (**B**) U-CDDP, (**C**) CS-CDDP, and (**D**) PLGA.
**Note:** Endotherms are indicated as peaks.
**Abbreviations:** DSC, differential scanning calorimetry; U, uniform; CS, core–shell; CDDP, cisplatin; PLGA, poly(lactic-co-glycolic acid).
