# Supplementary figures and images for: Electrohydrodynamic fabrication of core–shell PLGA nanoparticles with controlled release of cisplatin for enhanced cancer treatment
Source: Int J Nanomedicine. 2017 May 23;12:3913–26. doi: 10.2147/IJN.S134833 (PMC5449170; doi:10.2147/IJN.S134833)

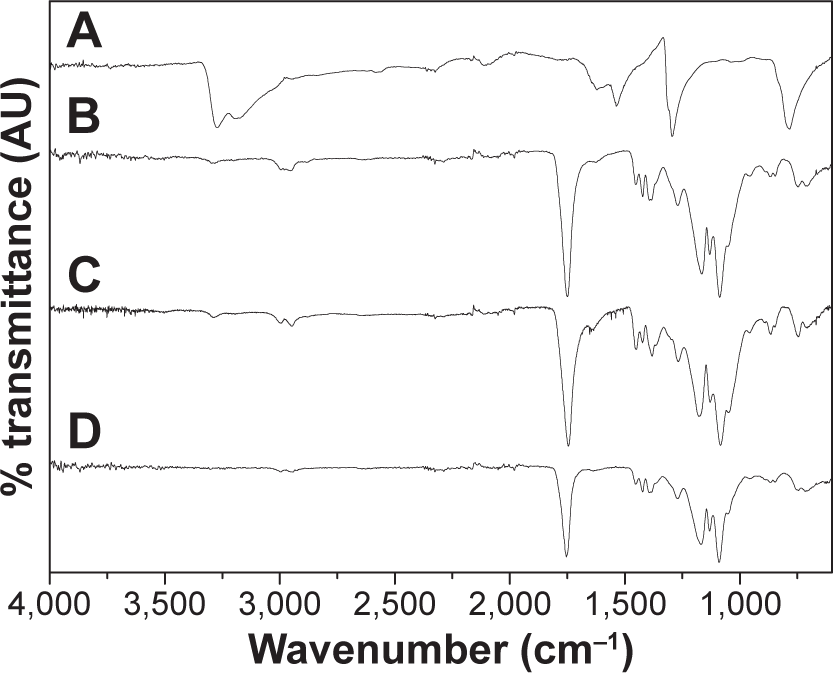

Supplement: Figure S1 — FTIR spectra of (A) CDDP, (B) U-CDDP, (C) CS-CDDP, and (D) PLGA. Abbreviations: AU, arbitrary unit; FTIR, Fourier transform infrared; U, uniform; CS, core–shell; CDDP, cisplatin; PLGA, poly(lactic-co-glycolic acid). [file ijn-12-3913s1.tif]

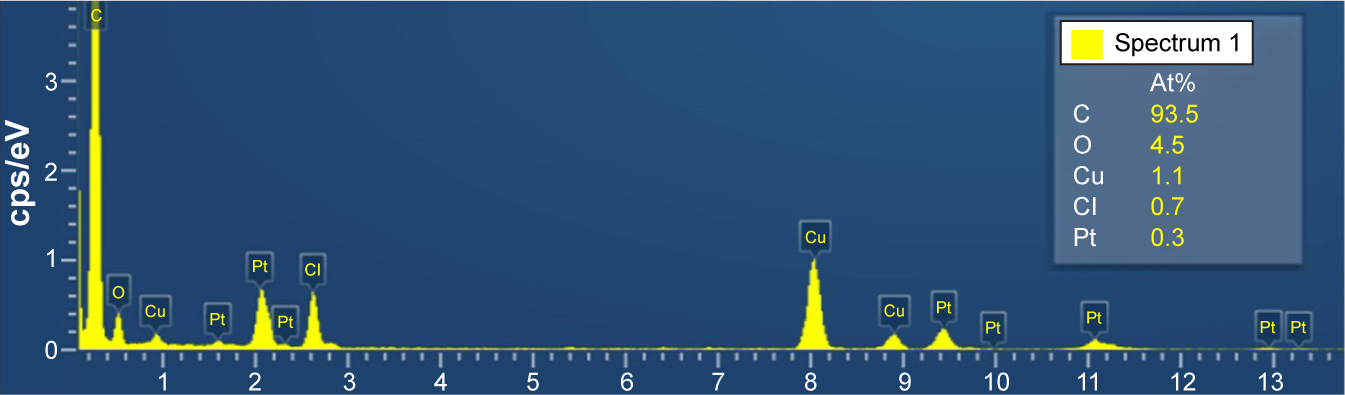

Supplement: Figure S2 — Typical EDS spectra of CDDP region of CS-CDDP NPs. Abbreviations: EDS, energy-dispersive X-ray spectroscopy; CS, core–shell; CDDP, cisplatin; NPs, nanoparticles; At%, atomic percent (the percentage of one kind of atom relative to the total number of atoms). [file ijn-12-3913s2.tif]

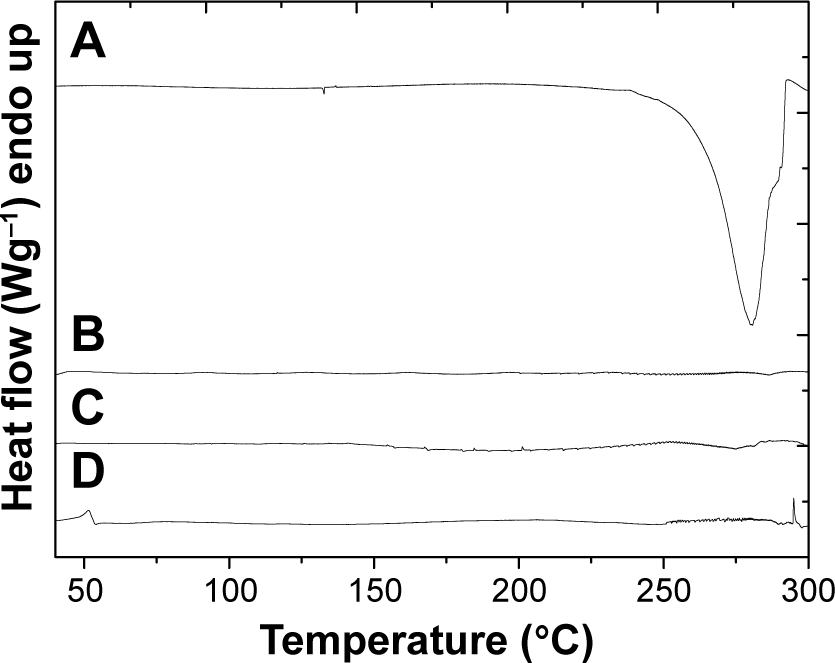

Supplement: Figure S3 — DSC thermogram of (A) CDDP, (B) U-CDDP, (C) CS-CDDP, and (D) PLGA. Note: Endotherms are indicated as peaks. Abbreviations: DSC, differential scanning calorimetry; U, uniform; CS, core–shell; CDDP, cisplatin; PLGA, poly(lactic-co-glycolic acid). [file ijn-12-3913s3.tif]
